# cmFPA008, an anti-mouse CSF-1R antibody, combines with multiple immunotherapies to reduce tumor growth in nonclinical models

**DOI:** 10.1186/2051-1426-3-S2-P351

**Published:** 2015-11-04

**Authors:** David Bellovin, Nebiyu Wondyfraw, Anita Levin, David G DeNardo, Emma Masteller, Thomas Brennan

**Affiliations:** 1 Five Prime Therapeutics Inc., South San Francisco, CA, USA; 2 Washington University St. Louis, School of Medicine, St. Louis, MO, USA

## 

The colony stimulating factor 1 receptor (CSF1R) signaling pathway drives the recruitment, differentiation, and survival of tumor-associated macrophages (TAMs) in the neoplastic microenvironment, promoting tumor progression and modulation of the immune response. As such, CSF1R represents an intriguing therapeutic target for immuno-oncology. FivePrime has developed FPA008, an IgG4 antibody with high affinity (K_D_ = 0.35 nM) for CSF1R and the ability to block binding of both CSF1 and IL-34 to this receptor. In order to interrogate the impact of CSF1R signaling inhibition on tumor growth and the immune environment, we generated a surrogate antibody, cmFPA008, which targets mouse CSF1R and demonstrates equivalent affinity and ligand-blocking ability as FPA008.

Consistent with other reports on molecules targeting this pathway, cmFPA008 as monotherapy results in statistically significant but modest growth inhibition in multiple preclinical tumor models, including MC38 colon adenocarcinoma and B16 melanoma. Utilizing a combination of flow cytometric, immunohistochemical, and gene expression analyses, we show that CSF1R inhibition induces dramatic reduction of TAMs and an increase in the CD8+ T cell to regulatory T cell ratio in syngeneic tumor models.

Given that the regulation an anti-tumor immune response is complex, effective cancer therapy may require combining multiple immunotherapy agents. We sought to determine whether inhibition of CSF1R when combined with other immuno-oncology therapeutics enhanced the anti-tumor impact. We observed increased PD-L1 (CD274) expression in the tumor after treatment with cmFPA008 monotherapy, thereby providing a rationale for a combination of FPA008 with a compound targeting the PD-1 pathway. Our results show that cmFPA008 significantly enhances anti-tumor efficacy when combined with an anti-PD1 therapeutic in multiple syngeneic tumor models. In addition, we show that co-administration of cmFPA008 with an agonist anti-CD40 significantly enhances tumor suppression compared to either therapy alone. Changes in tumor-infiltrating lymphocyte (TIL) populations upon treatment provide important insight into the mechanism of action of cmFPA008 monotherapy and in combination with other immunotherapies.

These results provide support for ongoing clinical efforts to evaluate FPA008 as an anti-cancer immunotherapy, particularly in combination with other immuno-oncology therapeutics. FivePrime has planned a clinical trial in collaboration with Bristol-Myers Squibb (BMS) to investigate the efficacy of FPA008 in combination with the anti-PD1 therapeutic Opdivo^®^ (nivolumab) in six tumor types.

